# The top three causes of prematuremortality in Belgrade 2020: Milena Šantrić Milićević

**DOI:** 10.1093/eurpub/ckac129.547

**Published:** 2022-10-25

**Authors:** N Rosić, M Šantrić Milićević, A Stevanović, V Bjegović Mikanović, Z Terzić Šupić, J Todorović, N Terzić, S Stojisavljević, T Albreht

**Affiliations:** 1 City Institute of Public Health Belgrade, Belgrade, Serbia; 2 Institute of Social Medicine, Faculty of Medicine, University of Belgrade, Belgrade, Serbia; 3 Center – School of Public Health, Faculty of Medicine, University of Belgrade, Belgrade, Serbia; 4 Center for Health System Development, Institute of Public Health of Montenegro, Pogorica, Montenegro; 5 Department of Social Medicine, Health Institute of the Republic of Srpska, Banja Luka, Bosnia and Herzegovina; 6 Department of Public Health, Faculty of Medicine, University of Banja Luka, Banja Luka, Bosnia and Herzegovina; 7 Center for Health Care, National Institute of Public Health Slovenia, Ljubljana, Slovenia

## Abstract

**Background:**

Analysis of years of life lost (YLL) due to premature deaths during the COVID-19 pandemic can direct decision-makers towards specific public health recommendations in order to improve health and lives of people. Our study aimed to examine the existence of age- and sex-specific patterns of the three most common causes of premature death in Belgrade during the first year of the COVID-19 epidemic.

**Methods:**

Mortality data disaggregated by age, sex and cause of death, as well as the estimated number of inhabitants and remaining life-expectancy by age-groups for Belgrade was provided by the Statistical Office of the Republic of Serbia. YLLs were calculated using the methods of the Global Burden of Disease Study, without garbage code redistribution. Mortality rates were standardized according to the European Standard Population. We acknowledge the support from the COST Action 18218 - European Burden of Disease Network.

**Results:**

In 2020 in Belgrade, according to the share in all-cause YLLs, cardiovascular diseases ranked first (36.2%), followed by neoplasms (25.7%) and COVID-19 (11.1%). However, on average, COVID-19 generated higher number of YLLs per death case (11.9) than cardiovascular diseases (9.2), but fewer than neoplasms (13.9). In total of 31,943 YLLs due to COVID-19, men had 1.7 times more YLLs than women. By age groups, the highest YLL share due to COVID-19 was among men aged 45-49 (16%) and 70-74 (16%) and among women aged 20-25 (33%) and 25-29. years (29%). In men, COVID-19 YLL rate was 2,488 per 100,000 and was higher after standardization (2,714). In women, COVID-19 YLL rate was 1346 per 100,000 and was lower after standardization (1,245).

**Conclusions:**

In Belgrade, COVID-19 was the third cause of premature mortality in 2020. The difference between COVID-19 YLL rates in men and women were even more prominent after standardization. Future research is needed to determine the synergistic impact of COVID-19 and other causes of premature death.

**Key messages:**

• In 2020, COVID-19 was among the top three causes of premature mortality among male and female contingents of the Belgrade population.

• Assessing the causes of premature mortality is important for determining community health priorities.

